# Statistical Power to Detect Genetic (Co)Variance of Complex Traits Using SNP Data in Unrelated Samples

**DOI:** 10.1371/journal.pgen.1004269

**Published:** 2014-04-10

**Authors:** Peter M. Visscher, Gibran Hemani, Anna A. E. Vinkhuyzen, Guo-Bo Chen, Sang Hong Lee, Naomi R. Wray, Michael E. Goddard, Jian Yang

**Affiliations:** 1 The University of Queensland, Queensland Brain Institute, Brisbane, Queensland, Australia; 2 The University of Queensland Diamantina Institute, The Translational Research Institute, Brisbane, Queensland, Australia; 3 University of Melbourne, Department of Food and Agricultural Systems, Parkville, Victoria, Australia; 4 Biosciences Research Division, Department of Primary Industries, Bundoora, Victoria, Australia; Stanford University School of Medicine, United States of America

## Abstract

We have recently developed analysis methods (GREML) to estimate the genetic variance of a complex trait/disease and the genetic correlation between two complex traits/diseases using genome-wide single nucleotide polymorphism (SNP) data in unrelated individuals. Here we use analytical derivations and simulations to quantify the sampling variance of the estimate of the proportion of phenotypic variance captured by all SNPs for quantitative traits and case-control studies. We also derive the approximate sampling variance of the estimate of a genetic correlation in a bivariate analysis, when two complex traits are either measured on the same or different individuals. We show that the sampling variance is inversely proportional to the number of pairwise contrasts in the analysis and to the variance in SNP-derived genetic relationships. For bivariate analysis, the sampling variance of the genetic correlation additionally depends on the harmonic mean of the proportion of variance explained by the SNPs for the two traits and the genetic correlation between the traits, and depends on the phenotypic correlation when the traits are measured on the same individuals. We provide an online tool for calculating the power of detecting genetic (co)variation using genome-wide SNP data. The new theory and online tool will be helpful to plan experimental designs to estimate the missing heritability that has not yet been fully revealed through genome-wide association studies, and to estimate the genetic overlap between complex traits (diseases) in particular when the traits (diseases) are not measured on the same samples.

## Author Summary

Genome-wide association studies (GWAS) have identified thousands of genetic variants for hundreds of traits and diseases. However, the genetic variants discovered from GWAS only explained a small fraction of the heritability, resulting in the question of “missing heritability”. We have recently developed approaches (called GREML) to estimate the overall contribution of all SNPs to the phenotypic variance of a trait (disease) and the proportion of genetic overlap between traits (diseases). A frequently asked question is that how many samples are required to estimate the proportion of variance attributable to all SNPs and the proportion of genetic overlap with useful precision. In this study, we derive the standard errors of the estimated parameters from theory and find that they are highly consistent with those observed values from published results and those obtained from simulation. The theory together with an online application tool will be helpful to plan experimental design to quantify the missing heritability, and to estimate the genetic overlap between traits (diseases) especially when it is unfeasible to have the traits (diseases) measured on the same individuals.

## Introduction

Genome-wide association studies (GWAS) have been extremely successfully in identifying genetic variants associated with complex traits and diseases in humans [1]. In GWAS, hundreds of thousands or millions of SNPs are tested one by one for statistical evidence of association with a trait, and to avoid false positive discoveries due to the very large number of statistical tests being conducted, usually a very stringent p-value threshold, e.g. 5×10^−8^, is used to report a significant finding. Therefore, if there are many genes each with a small effect affecting the trait, most of these genetic variants will fail to pass the stringent threshold and remain undetected. This is one of the explanations of the ‘missing heritability’ question, that genetic variants identified from GWAS so far explain a fraction of the heritability for complex traits [2]. We proposed a method, which is able to estimate the total amount of variance explained by all SNPs together without testing the SNPs individually for a quantitative trait [3], and subsequently extended it to the estimation of missing heritability for binary disease data from ascertained case-control studies [4]. The analyses until recently only included common SNPs (e.g. minor allele frequency >0.01). The estimate quantifies the overall contribution from the additive effects of all SNPs, which is the upper limit of the proportion of variance that is captured by the additive effects of the set of SNPs used in the estimation, and is also the lower limit of the narrow-sense heritability of the trait. We also extended the method to estimate the genetic correlation between two traits using SNP data [5], [6]. In contrast to the traditional (co)variance estimation methods that rely on pedigree information (family/twin studies), our method uses unrelated samples from a general population and the genetic (co)variance is estimated using a genetic relationship matrix (GRM) estimated from SNPs. The estimate of genetic variance using SNP data in unrelated individuals is free of confounding from common environment effects shared between close relatives that are difficult to model in family-based analyses, and is directly comparable to results from GWAS, because both are based on the same experimental design. For multiple trait analysis, the SNP-based approach allows the estimation of the genetic correlation between complex traits measured on different samples [6], [7]_ENREF_8. This is important in particular for estimating the genetic correlation between diseases because multiple diseases are unlikely to co-segregate in sufficiently large pedigrees to allow estimation using traditional pedigree design. The SNP-based method has the flexibility of estimating the genetic correlation between any two diseases using completely independent case-control data. Other methods to estimate genetic parameters from individual-level or summary GWAS data have also been reported [8]–[10].

We previously named the SNP-based method mentioned GREML [11], as a complement to GBLUP [12] where variance components are assumed known, and have been implemented them in the software tool GCTA [13]. One outstanding question is the statistical power of detecting genetic variation using the population-based estimation method, for example how many samples are required to achieve estimates that are sufficiently accurate to detect genetic (co)variance of complex traits. In this paper, we derive the sampling variance of the estimate of genetic (co)variance by analytical derivations and verify our derivations by simulations under a range of scenarios. We also provide an online tool for power calculation.

## Methods and Results

### Univariate analysis

The methods of using SNP data to estimate genetic variance in unrelated individuals have been detailed elsewhere [3], [13]. In brief, given GWAS data, we can model the phenotype as
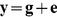
(1)where **y** is an *N*×1 vector of phenotypes with *N* being the sample size, **g** is an *N*×1 vector with each of its elements being the total genetic effect of an individual captured by all SNPs, and **e** is an *N*×1 vector of residuals. We have 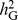 and 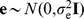, where 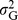 is the genetic variance captured by all SNPs, **A** is the genetic relationship matrix (GRM) estimated from SNPs [3], 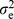 is the residuals variance and **I** is an identity matrix. The genetic relationships, also known as ‘genomic relationships’ or ‘genetic similarity relationships’, are referenced to the current population, and so can be negative as they are distributed about a mean of zero. Equation (1) is a typical mixed linear model with 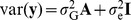, in which the variance components can be estimated using a restricted maximum likelihood (REML) approach [13], [14]. The proportion of variance explained by all SNPs (SNP heritability) is defined as 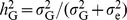.

For power calculation, we need to know the sampling variance of the estimate of 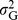, i.e. 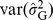. In practice, the asymptotic sampling variance (standard error squared) of a variance component is calculated from a diagonal element of the inverse of the information matrix in maximum likelihood analysis [15]–[18]. Each element of the information matrix, however, comprises complex forms of matrix algebra including a matrix inverse. It is therefore unfeasible to derive 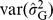 directly from the inverse of the information matrix. We show below an equivalent approach to obtain 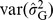 under the simple regression framework.

**Figure 1:**
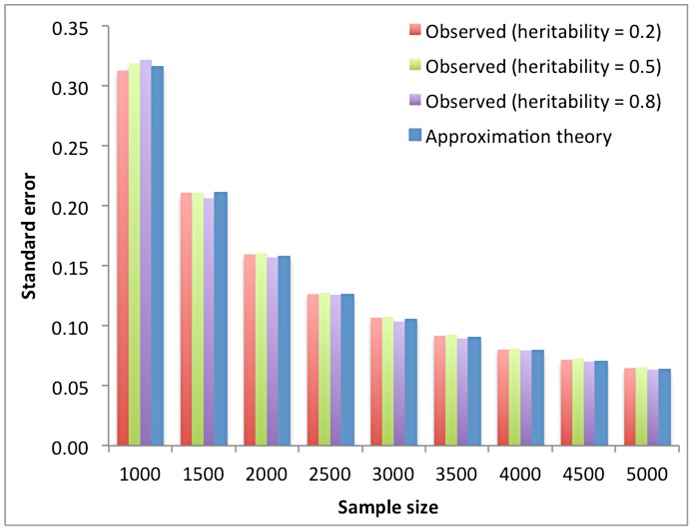
Standard error of the estimate of variance explained by all SNPs vs. sample size. The first three columns are the averaged standard error observed from 100 simulations under three heritability levels. The last column is the predicted standard error from our approximation theory. The plotted data can be found in Table S1.

For unrelated individuals, where the phenotypic correlation between individuals is small, mixed linear model analysis using the REML approach is asymptotically equivalent to simple regression analysis of pairwise phenotypic similarity/difference on pairwise genetic similarity, as measured by identity-by-descent (IBD) or identity-by-state (IBS) at genome-wide markers [17]–[20]. Under such circumstance, a regression of the cross-product of the phenotypes is equivalent to using both the squared difference and squared sum of the pairwise phenotypes, and using the cross-product is equivalent to using maximum likelihood [19]. The model for the regression-based analysis can be written as
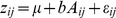
(2)where 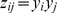 with 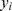 and 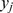 being the phenotypes of individuals *i* and *j* (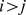), 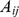 is the *ij*-th element of the GRM **A**, and 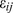 is the residual of this regression. There are 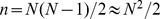 observations (contrasts) in the regression. The regression coefficient *b* is equivalent to 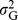 because
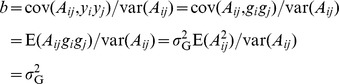
In such a simple regression, the sampling variance of the estimate of the regression coefficient is
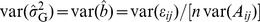
(3)If the samples are unrelated and the phenotypes have been standardized with mean of 0 and variance of 1, then 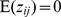 and 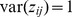. Since 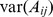 is small, there is hardly any variance in 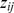 that can be explained by 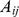 so that 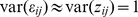. We therefore have
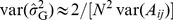
(4)Under circumstances when 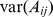 is large, for example when the GRM is calculated from pedigree data, a substantial proportion of variance in 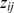 could be explained by 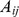, so that 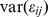 will be smaller than 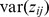 and the sampling variance of estimate of genetic variance will be reduced accordingly. In general, 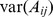 and the residual variance in equation (2) depend on the number of SNP that are used to calculate the GRM and their correlation structure. Although 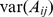 can be calculated empirically from the data, theoretical work suggest it is approximately 2×10^−5^ for genome-wide coverage of common SNPs in human populations [21]. Since the phenotypic variance is usually estimated with very high precision,
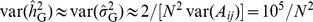
(5)This suggests that the standard error (SE) of 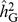 depends only on sample size, and is approximately 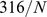. We show by simulations based on real genotype data (Text S1) that this approximation is very accurate (Figure 1 and Table S1). The SEs calculated from the approximation theory are also highly consistent with those reported from our previous studies for human height and body mass index (BMI). For example, the reported SE of 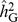 for height was 0.083 using 3925 unrelated samples [3] and 0.029 for both height and BMI, irrespective to 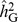, using 11586 unrelated samples [22], and the SE calculated from the approximation theory is 0.081 for *N* = 3925 and 0.027 for *N* = 11586.

### Bivariate analysis (traits measured on the same individuals)

For a bivariate analysis where the two traits are measured on the same individuals, the mixed linear model can be written as [6]

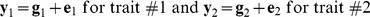
(6)where **y**_1_ and **y**_2_ are *N*×1 vector of phenotypes, **g**_1_ and **g**_2_ are *N*×1 vectors of genetic effects with 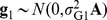 and 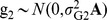, **e**_1_ and **e**_2_ are *N*×1 vectors of residuals with 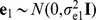 and 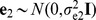, and *N* is the sample size. The variance covariance matrix is
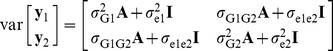
where 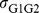 is the genetic covariance between the two traits and 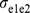 is the residual covariance. The genetic variance and covariance components can also be estimated using REML [6]. The genetic correlation is estimated as 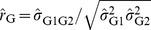. Since 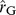 is a non-linear function of 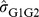, 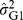 and 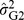, there is no explicit derivation for 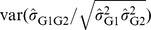. Reeve (1955) and Robertson (1959) provided an approximation of 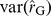 in the context of balanced pedigree design as 
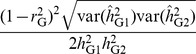
 [23], [24] and Koots and Gibson (1996) proposed a modified version as 
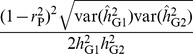
 [25], where 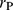 is the phenotypic correlation between the traits. However, both approximations have an unsatisfying property that 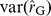 will approach 0 if 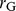 or 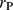 is close to 1. We derived an approximation, which does not have this problem (Text S2), i.e.
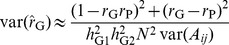
(7)As described above, 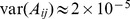 for a GRM estimated from common SNPs in unrelated individuals in human populations, therefore 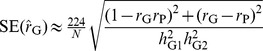. When 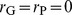, i.e. the traits are completely independent, 
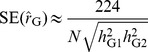
. We tested equation (7) by simulations based on real genotype data (Text S1). The simulation results suggest that the approximation is reasonably accurate (Table 1). For real data analysis, we previously estimated the genetic correlation between intelligence at age 11 years and in old age of 0.62 with a SE of 0.23 using 1729 samples [5], consistent with the predicted SE of 0.22 from the approximation theory.

**Table 1:** Standard error of the estimate of genetic correlation from a bivariate analysis of two traits measured on the same or different samples using genome-wide SNP data.

	Same sample					Different samples					
*r*_G_	*N*	Est.	SE (Obs.)	s.e.m.	SE (Approx.)	*N*_1_	*N*_2_	Est.	SE (Obs.)	s.e.m.	SE (Approx.)
**0.0**	4000	0.00	0.128	0.0024	0.114	1000	3000	0.04	0.288	0.0126	0.264
**0.0**	6000	0.00	0.085	0.0009	0.076	2000	4000	0.00	0.191	0.0062	0.161
**0.0**	8000	−0.01	0.065	0.0006	0.057	3000	5000	−0.01	0.129	0.0021	0.118
**0.0**	10000	0.00	0.053	0.0004	0.046	4000	6000	−0.01	0.103	0.0014	0.093
**0.4**	4000	0.42	0.112	0.0019	0.108	1000	3000	0.32	0.295	0.0124	0.309
**0.4**	6000	0.39	0.076	0.0009	0.072	2000	4000	0.39	0.230	0.0141	0.182
**0.4**	8000	0.38	0.057	0.0006	0.054	3000	5000	0.37	0.136	0.0036	0.131
**0.4**	10000	0.39	0.046	0.0005	0.043	4000	6000	0.38	0.107	0.0015	0.103
**0.8**	4000	0.80	0.081	0.0024	0.091	1000	3000	0.62	0.417	0.0274	0.418
**0.8**	6000	0.80	0.056	0.0017	0.061	2000	4000	0.83	0.248	0.0127	0.232
**0.8**	8000	0.80	0.042	0.0012	0.046	3000	5000	0.86	0.198	0.0069	0.164
**0.8**	10000	0.79	0.036	0.0010	0.036	4000	6000	0.83	0.133	0.0034	0.127

Same sample: two traits are measured on the same set of samples. Different sample: two traits are measured on the different sets of samples. 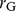: parameter of genetic correlation (i.e. proportion of simulated causal variants shared between the two traits). Est.: estimate of genetic correlation from 100 simulations. SE(Obs.): mean of the observed standard errors from 100 simulations. s.e.m.: standard error of the mean (i.e. SE(Obs.)). SE(Approx.): standard error calculated from our approximation theory.

### Bivariate analysis (traits measured on different sets of individuals)

For a bivariate analysis where the two traits are measured on different sets of individuals, e.g. height in males and blood pressure in females, the variance-covariance matrix is [6]

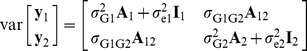
where **y**_1_ is an *N*_1_×1 vector of phenotypes in sample set #1 (e.g. males), and **y**_2_ is an *N*_2_×1 vector of phenotypes for in sample set #2 (e.g. females), with *N*_1_ and *N*_2_ being the sample sizes of the two sets. **A**_1_ is an *N*_1_×*N*_1_ GRM for individuals in sample set #1, **A**_2_ is an *N*_2_×*N*_2_ GRM in sample set #2 and **A**_12_ is an *N*_1_×*N*_2_ GRM between the two sets of samples. 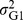 and 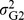 are the genetic variance for the two traits. 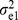 and 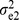 are the residual variances with the corresponding identify matrix **I**_1_ and **I**_2_. 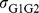 is the genetic covariance between traits. Since the two traits are measured on different sets of samples, the residual covariance is ignored because it is assumed that there is no covariance between the unrelated individuals apart from that caused by genetic factors. The genetic correlation is also estimated as 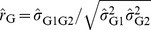, however, the sampling variance of 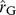 is different from that described above. Since the traits are measured in different sets of samples, 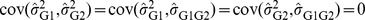. Therefore, from a second order Taylor series approximation [15]

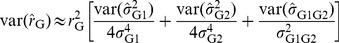
(8)This approximation involves the sampling variance of 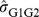. We show below an equivalent approach to obtain 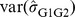.

Analogous to the univariate analysis, estimation of genetic covariance by a bivariate mixed linear model analysis is asymptotically equivalent to the following linear regression model
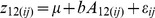
(9)where 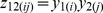 i.e. the product of phenotypes between the *i*-th individual in set #1 and the *j*-th individual in set #2, and 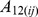 is the *ij*-th element of the GRM **A**_12_, i.e. the genetic relationship between the *i*-th individual in set 1 and the *j*-th individual in sample set #2. The regression coefficient is equivalent to genetic covariance between the two traits because
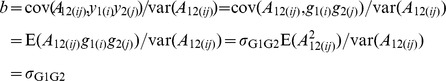
If the two sample sets are independent and phenotypes for both traits have been standardized with mean of 0 and variance of 1, then 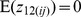 and 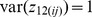. Since 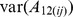 is small, 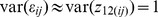. We then have 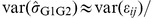 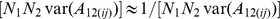.

We know from the derivations above that 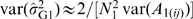 and 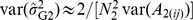. For unrelated individuals sampled from the same population, 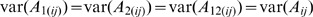, we therefore get
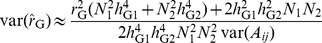
(10)This was also tested by simulations (Text S1) and the approximated standard errors were highly consistent with those observed from simulations, especially when sample size was large (Table 1). When 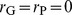, i.e. two traits are completely independent, 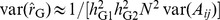 for traits measured on the same sample, and 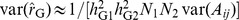 for traits measured on different samples. Therefore, for independent traits, the ratio of sampling variance of genetic correlation between the two traits measured on the same sample to that on different samples is simply 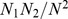.

### Case-control studies

For case-control studies, the proportion of variance in case-control status (0 or 1) that is explained by all SNPs on the observed scale (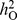) can be estimated using a linear model [4]. Therefore, the same approximations to the sampling variance of genetic variance and genetic correlation for quantitative traits can be applied directly to case-control studies. As shown in equation (5), in a univariate analysis, the sampling variance of SNP-based heritability depends only on sample size and variance in genetic relatedness, independent of the properties of the phenotype, so that var(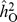) is also approximately 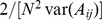 in a case-control study with *N* being the total number of cases and controls. We show in Table 2 that the observed standard errors of the estimates of 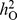 from published studies are highly consistent with those predicted from our approximation theory.

**Table 2:** Standard errors of the estimates of variance explained by all SNPs on the observed scale (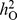) from published analyses of case-control studies for a number of diseases vs. those predicted from the approximation theory.

*Disease*	*N*_cases_	*N*_controls_	*Prevalence*	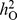	SE(Obs.)	SE(Approx.)
**Multiple sclerosis** [34]	1604	1953	0.001	0.851	0.088	0.089
**Alzheimer's disease** [34]	3290	3849	0.020	0.364	0.049	0.044
**Endometriosis** [34]	3154	6981	0.080	0.231	0.036	0.031
**Schizophrenia** [7]	9087	12171	0.010	0.410	0.015	0.015
**Bipolar disorder** [7]	6704	9031	0.010	0.441	0.021	0.020
**MDD** [7]	9041	9381	0.150	0.177	0.017	0.017
**ASD** [7]	3303	3428	0.010	0.310	0.046	0.047
**ADHD** [7]	4163	12040	0.050	0.253	0.020	0.020

*N*_cases_: number of cases. *N*_controls_: number of controls. SE(Obs.): reported standard error of the estimate of 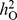 from real data analysis. SE(Approx.): standard error of 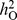 calculated from our approximation theory. MDD: major depression disorder. ASD: autism spectrum disorders. ADHD: attention-deficit/hyperactivity disorder.

**Figure 2:**
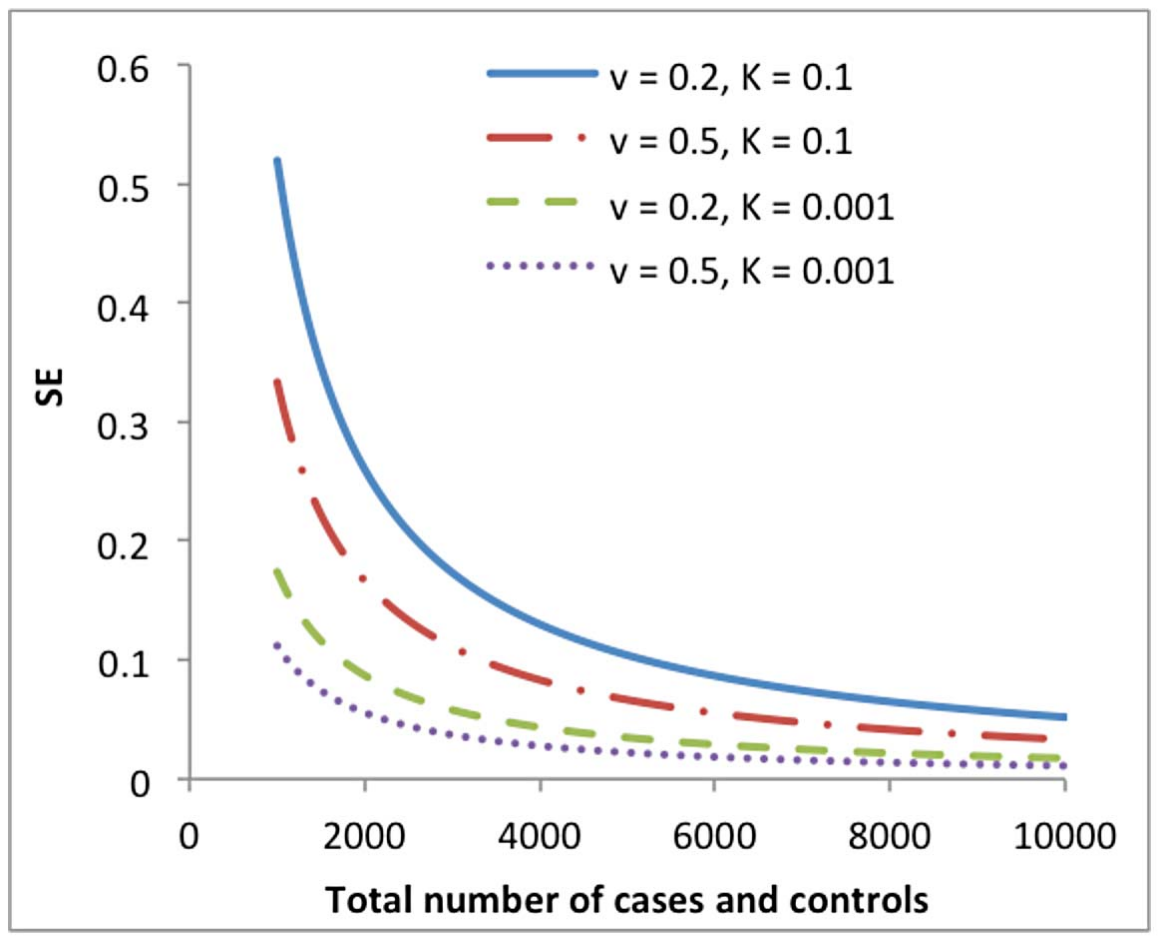
Standard error (SE) of the estimate of variance explained by all SNPs on the underlying scale (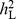) from a univariate analysis of a case-control study vs. total number of cases and controls (sample size). The SE is predicted from the approximation theory given different levels of disease prevalence (*K*) and proportion of cases in the sample (*v*).

To calculate power, however, we would need to specify 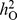, which is a parameter with non-intuitive properties, and depends on the prevalence of the disease in the population (*K*), the proportion of variance in disease liability that is captured by the SNPs at population level, and the proportional of cases in the sample (*v*). For this reason we define 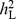 as the variance explained by all SNPs at the population level on the unobserved underlying scale of disease liability, and use a linear transformation to transform 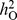 to 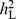 on the liability scale [4], i.e. 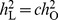 and 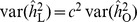 with 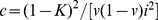. We then get
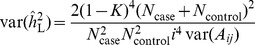
(11)where *N*_case_ is the number of cases, *N*_control_ is the number of controls, and *i* is the selection intensity which is a function of *K* [4]. We illustrate in Figure 2 the dependency of the SE of 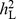 on disease prevalence (*K*) and proportion of cases in the sample (*v*) due to the transformation.

As shown in equation (10), in a bivariate analysis where traits are measured on different sets of samples, the sampling variance of genetic correlation depends on sample sizes, trait heritabilities and the genetic correlation parameter, which is also independent of the properties of the phenotypes. Therefore, in a bivariate analysis of two independent case-control disease studies,
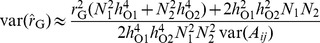
(12)where *N*_1_ and *N*_2_ are the total numbers of cases and controls of the two case-control studies, respectively. This also applies to a bivariate analysis of a quantitative trait and a cases-control disease study on different sets of samples, i.e.
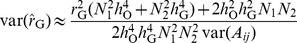
(13)These two equations can also be expressed with respect to 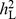, given 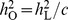 (see above). We show in Table 3 that the reported SEs of 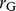 from bivariate analyses of psychiatric diseases are also highly in line with the predicted SEs from the approximation theory.

**Table 3:** Standard errors of the estimates of genetic correlations from published bivariate analyses of case-control studies for psychiatric diseases [7] vs. those predicted from the approximation theory.

Disease	*K*	*N*_cases_	*N*_controls_	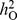	Disease	*K*	*N*_cases_	*N*_controls_	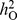	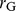	SE (Obs.)	SE (Approx.)
**SCZ**	0.01	9032	7980	0.40	**BPD**	0.01	6664	5258	0.39	0.68	0.044	0.049
**SCZ**	0.01	9051	10385	0.38	**MDD**	0.15	8998	7823	0.16	0.43	0.055	0.057
**SCZ**	0.01	9111	12146	0.41	**ASD**	0.01	3226	3308	0.29	0.16	0.059	0.057
**SCZ**	0.01	9013	10115	0.42	**ADHD**	0.05	4108	9936	0.22	0.08	0.046	0.045
**BPD**	0.01	6665	7408	0.42	**MDD**	0.15	8997	7680	0.17	0.47	0.061	0.063
**BPD**	0.01	6704	9030	0.43	**ASD**	0.01	3207	3294	0.31	0.04	0.065	0.061
**BPD**	0.01	6656	7041	0.38	**ADHD**	0.05	4099	9873	0.25	0.05	0.053	0.052
**MDD**	0.15	9031	9370	0.17	**ASD**	0.01	3239	3331	0.31	0.05	0.089	0.090
**MDD**	0.15	8936	8668	0.16	**ADHD**	0.05	4098	11233	0.24	0.32	0.071	0.073
**ASD**	0.01	3156	3254	0.27	**ADHD**	0.05	4181	12022	0.23	−0.13	0.087	0.090

SCZ: schizophrenia. BPD: bipolar disorder. MDD: major depression disorder. ASD: autism spectrum disorders. ADHD: attention-deficit/hyperactivity disorder. *N*_cases_: number of cases. *N*_controls_: number of controls. *K*: disease prevalence. 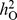: estimate of variance explained by all SNPs on the observed scale, which was calculated from the reported 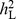 and disease prevalence in Supplementary Table 1 of Lee et al. [7]. 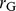: genetic correlation. SE(Obs.): reported standard error of the estimate of 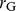 from real data analysis. SE(Approx.): standard error of 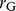 calculated from our approximation theory.

### Statistical power

**Figure 3:**
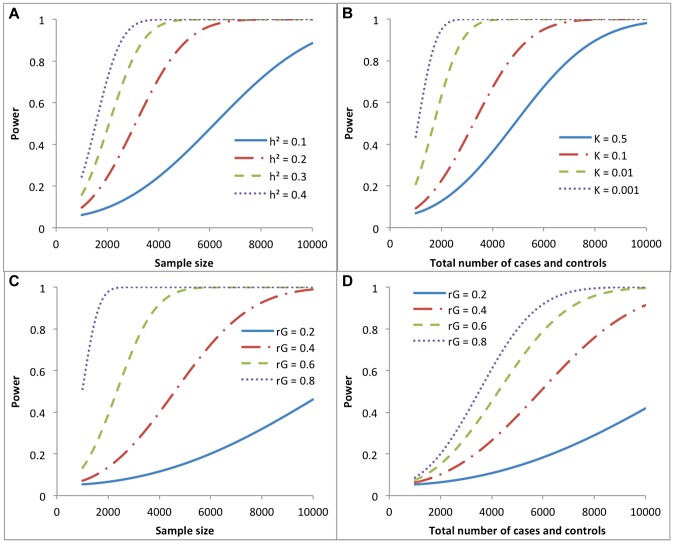
Statistical power of detecting genetic variance (correlation) under different study designs. **a**) Univarite analysis of a quantitative trait. **b**) Univariate analysis of a case-control study assuming equal number of cases and controls (*v* = 0.5) and heritability of liability (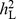) of 0.2. **c**) Bivariate analysis of two quantitative traits measured on the same set of individuals, assuming heritability of 0.2 for both traits. **d**) Bivariate analysis of two case-control studies on independent sets of samples, assuming equal numbers of cases and controls for each disease, and equal sample size (total number of cases and controls), equal heritability of liability (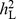 = 0.2) and equal prevalence (*K* = 0.01) for both diseases.

Statistical power is calculated from the population value of the parameter and its sampling variance, which was derived above. If the parameter is *θ*, where *θ* is either the proportion of phenotypic variance captured by SNPs (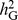) in the univariate case or the genetic correlation (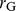) in the bivariate case, then 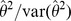 is asymptotically distributed as a non-central *χ* ^2^ with 1 degree of freedom and non-centrality parameter (NCP) of 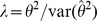. Given *λ* and the type-I error rate of *α*, statistical power is the probability that a non-central *χ* ^2^ variable is larger than the central *χ* ^2^ threshold that is determined by *α*. We show in Figure 3 the statistical power based on the sampling variance from our approximation theories to detect 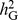 in a univariate case and 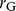 in a bivariate case under a range of scenarios. For example, for a quantitative trait, approximately 8900, 4500, 3000 and 2300 independent individuals are required to detect 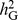 of 0.1, 0.2, 0.3 and 0.4 with >80% power at a type-I error rate of 0.05, respectively. For two quantitative traits measured on the same sample, approximately 7000, 4700, 2500 and 1600 independent individuals are required to detect 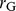 of 0.2, 0.4, 0.6 and 0.8 with >80% at a type-I error rate of 0.05, respectively.

### Online tool

We have also developed an online calculator (GCTA Power Calculator, http://spark.rstudio.com/ctgg/gctaPower), as part of the GCTA [13] software package (http://ctgg.qbi.uq.edu.au/software/gcta), using R-Shiny (http://shiny.rstudio.org) to calculate the SE of genetic variance or genetic correlation and statistical power given user-defined parameters.

## Discussion

We have derived the approximate sampling variance of the estimate of variance explained by all common SNPs (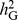) for a quantitative trait or case-control study of a disease, and genetic correlation (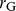) between two quantitative traits, between two diseases, or between a quantitative trait and a disease, using genome-wide SNP data in unrelated individuals. We believe that the derivations and the online tool will be helpful for researchers to determine how many samples are required to detect 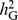 (or 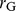) and to estimate 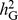 (or 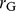) with adequate precision before collecting the genotype data.

The sampling variance of 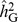 for a complex trait is inversely proportional to sample size (*N*) and the variance in SNP-based genetic relatedness (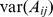), and independent of 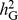. The sampling variance of 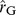 between two complex traits is a function of 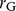, *N* of the two samples, 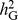 of the two traits and 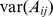 when the traits are measured on different samples, and further depends on the phenotypic correlation (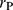) when traits are measured on the same samples. All the approximation theories apply to case-control studies of diseases since the case-control data can be analysed using a linear model on the observed 0–1 scale. The sampling variance for the estimate on the observed scale can then be transformed to that on the underlying liability scale using well-established theory. The standard errors (square root of sampling variance) of either 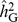 or 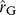 observed in published studies were all highly consistently with those predicted from our approximation theories, which were also confirmed by simulations based on real genotype data.

Analytical expressions for the sampling variance of the estimates of genetic (co)variance from pedigree analyses have been around for over 50 years [17], [26], and statistical power can be derived from these by using the sampling variance and population value of the parameter. However, these expressions are typically for specific structured pedigrees, such as fullsib or halfsib families or twin pairs. There are to our knowledge no simple approximations for general pedigrees, because the inverse of the variance-covariance matrix is required and this is conditional on the actual pedigree structure. The sampling variance of the estimated parameters in a general complex pedigree is usually derived post hoc after the analysis has been performed.

Methods for calculating the power of detecting quantitative trait loci (QTL) in family-based linkage studies have been investigated extensively in the past two decades [16]–[18], [27]. These methods were developed to calculate the power of detecting a QTL but can be generalized for variance components estimation, e.g. estimating the genetic variance using pedigree information. The non-centrality parameter of the test-statistic from a maximum likelihood analysis of variance components is 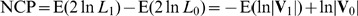, where *L* is the likelihood function, and 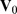 and 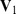 are the variance covariance matrix under the null and alternative hypotheses respectively [17], [18]. For a specific balanced pedigree design, e.g. fullsibs or nuclear families, the determinant (or inverse) of the **V** matrix can be computed explicitly, so that the NCP can be calculated without making approximation [16], [17]. For an arbitrary pedigree, 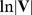 can be calculated approximately using Taylor expansions given the variance in family relatedness [18], [27]. Therefore, all these methods explicitly or implicitly require a known pedigree. When the correlations between relatives are small, the first order approximation of the NCP in Rijsdijk et al [18] can be written in our notations as 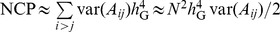, which is the same as we derived (i.e. 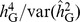, see Equation (4) for 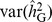), even though our deviations were based on least squares regression analysis in unrelated samples whereas the derivations in Rijsdijk et al [18] were based on maximum likelihood approach in family data. This approximation is reasonably accurate when correlations between relatives are small for a pedigree-based design, which is not an issue for a population-based design where the genetic relationships between unrelated samples are very small as demonstrated in Yang et al [3]. We show by simulations (Text S1) that for a univariate analysis the LRT statistics calculated based on REML are highly consistent with the chi-squared test-statistics calculated by the Wald test using the sampling variance either observed from the simulations or predicted from our approximation theory (Figure S1).

For a given population, a set of common SNPs and the method of calculating the genetic relationship matrix that we have used here, 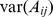 is a fixed quantity because it depends only on effective population size of the human populations [28]. We used 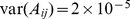, which was calculated from theory based upon an effective population size of 10,000. Variance in genetic relatedness (and therefore power of detection) can decrease by including many rare SNPs in calculating the GRM because adding more rare SNPs increases the effective population size reflecting recent population expansion. The variance in relatedness can also increase by sampling closer relatives (see below for more discussion) or, for example, by creating a relationship matrix based upon haplotype information. Modifying the GRM can also affect the variance of the off-diagonal elements. For example by applying a weighting of SNPs depending on linkage disequilibrium the variance in the estimates of genetic relationships will decrease so that the sampling variance of the estimate of SNP-based heritability will be increased [29]. Although we derive the theory and show the results based on the SNPs on the whole genome, our approximation theories are also applicable in analyses using a subset of SNPs, e.g. SNPs from a single chromosome. In that case, 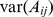 used in the approximation equations should be either observed empirically from data or derived from theory [28] based on the subset of SNPs.

If there are unknown related samples in the data (cryptic relatedness), 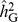 will possibly be inflated due to shared environment between close relatives and/or the effects of causal variants in LD with the SNPs but captured by family relatedness, and 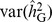 will be deflated due to the increase of 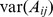. In fact, the interpretation of 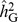 changes if there is a substantial proportion of close relatives in the data [30], [31]. This, however, affects GWAS result in a similar way, where the SE of the estimate of a SNP effect from a single SNP analysis (e.g. linear regression for a quantitative trait and logistic regression for a case-control study) will be deflated, causing an inflation of the test-statistics GWAS (often called “genomic inflation” [32]). For the estimation of 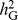 using all common SNPs, to avoid possible confounding from shared environments and uncaptured causal variants, we suggested in Yang et al. (2010) a stringent threshold, i.e. 0.025, to remove cryptic relatedness from the data so that the estimate of 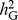 can be compared directly to the results from GWAS in response to the “missing heritability” problem [2]. In practice, observing a much smaller SE of 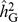 using all common SNPs than that predicted from theory is a caveat suggesting substantial cryptic relatedness remaining in the data.

Using the same experimental design of a sample of conventionally unrelated individuals, the experimenter can increase power by increasing sample size. Fortunately, power increases quadratically with sample size because every new sample is contrasted with all existing samples. The sampling variance of the estimate of the genetic correlation is generally much larger than that of the proportion of variance explained from a univariate analysis, consistent with the theory of the sampling variance of genetic correlations in pedigree designs [33].

## Supporting Information

Figure S1 Likelihood ratio test (LRT) statistic vs. Chi-squared test-statistic in a univariate analysis.
(PDF)

Table S1Standard error of the estimate of 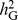 (variance explained by all SNPs) observed from 100 simulations vs. that calculated from our approximation theory.(PDF)

Text S1 Simulations.
(PDF)

Text S2 Sampling variance of genetic correlation.
(PDF)

Text S3 Acknowledgments to dbGaP data.
(PDF)
