# Supplementary material for: Statistical Power to Detect Genetic (Co)Variance of Complex Traits Using SNP Data in Unrelated Samples
Source: PLoS Genet. 2014 Apr 10;10(4):e1004269. doi: 10.1371/journal.pgen.1004269 (PMC3983037; doi:10.1371/journal.pgen.1004269)

Figure S1 Likelihood ratio test (LRT) statistic vs. Chi-squared test-statistic in a univariate analysis. $\text{LRT} = 2(\ln L_1 - \ln L_0)$ with L_0 and L_1 being the log-likelihood function of REML evaluated under the null and alternative hypotheses respectively, and $\chi^2 = \hat{h}_G^4 / \text{var}(\hat{h}_G^2)$ with the red dots representing χ^2 values calculated based on the observed sampling variance in the simulation and the blue crosses representing χ^2 values calculated based on the predicted sampling variance from our approximation theory. The simulations were performed at three levels of heritability, panel a) $h_G^2 = 0.2$, panel b) $h_G^2 = 0.5$ and panel c) $h_G^2 = 0.8$ (see Text S1 for details of the simulation design). Each plotted value is an average from 100 simulations.

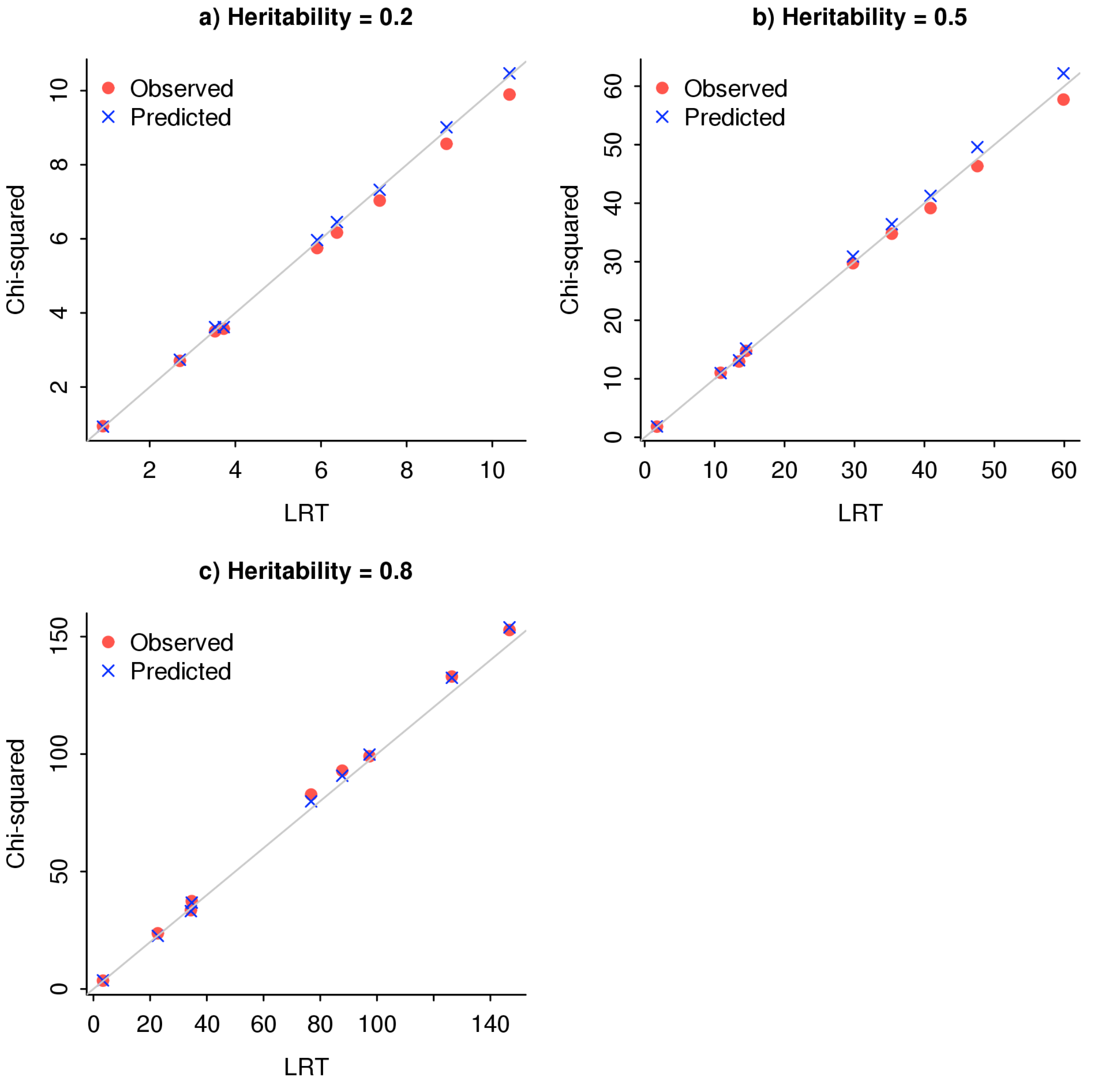

Supplement: Figure S1 — Likelihood ratio test (LRT) statistic vs. Chi-squared test-statistic in a univariate analysis. (PDF) [file pgen.1004269.s001.pdf]
