# Supplementary material for: Statistical Power to Detect Genetic (Co)Variance of Complex Traits Using SNP Data in Unrelated Samples
Source: PLoS Genet. 2014 Apr 10;10(4):e1004269. doi: 10.1371/journal.pgen.1004269 (PMC3983037; doi:10.1371/journal.pgen.1004269)
Supplement: Table S1 — Standard error of the estimate of (variance explained by all SNPs) observed from 100 simulations vs. that calculated from our approximation theory. (PDF) [file pgen.1004269.s002.pdf]

Table S1 Standard error of the estimate of h_G^2 (variance explained by all SNPs) observed from 100 simulations vs. that calculated from our approximation theory.

N	h_G^2 **= 0.2**		h_G^2 **= 0.5**		h_G^2 **= 0.8**		**SE (Approx.)**
	**SE (Obs.)**	***s.e.m.***	**SE (Obs.)**	***s.e.m.***	**SE (Obs.)**	***s.e.m.***	
500	0.63	2.6E-03	0.63	2.3E-03	0.63	1.3E-03	0.63
1000	0.31	9.8E-04	0.32	8.0E-04	0.32	4.6E-04	0.32
1500	0.21	5.5E-04	0.21	3.8E-04	0.21	2.4E-04	0.21
2000	0.16	3.6E-04	0.16	2.6E-04	0.16	1.9E-04	0.16
2500	0.13	2.6E-04	0.13	1.9E-04	0.13	1.1E-04	0.13
3000	0.11	1.9E-04	0.11	1.4E-04	0.11	9.9E-05	0.11
3500	0.09	1.9E-04	0.09	1.1E-04	0.09	8.8E-05	0.09
4000	0.08	1.4E-04	0.08	8.9E-05	0.08	6.6E-05	0.08
4500	0.07	1.3E-04	0.07	7.1E-05	0.07	5.9E-05	0.07
5000	0.06	9.7E-05	0.07	4.6E-05	0.06	5.7E-05	0.06

N: sample size. h_G^2 : heritability parameter used in simulations. SE(Obs.): mean of the observed standard errors from 100 simulations. s.e.m.: standard error of the mean (i.e. SE(Obs.)). SE(Approx.): standard error calculated from our approximation theory.
