# Supplementary material for: Statistical Power to Detect Genetic (Co)Variance of Complex Traits Using SNP Data in Unrelated Samples
Source: PLoS Genet. 2014 Apr 10;10(4):e1004269. doi: 10.1371/journal.pgen.1004269 (PMC3983037; doi:10.1371/journal.pgen.1004269)
Supplement: Text S1 — Simulations. (PDF) [file pgen.1004269.s003.pdf]

Text S1: Simulations

We performed simulation studies based on real genotype data accessed through dbGaP. The dataset consists of the ARIC (Atherosclerosis Risk in Communities), NHS (Nurses' Health Study) and HPFS (Health Professionals Follow-up Study) cohorts [1-3]. Descriptive summary and quality controls (QC) of the data have been detailed elsewhere [4]. In brief, there were 14,347 samples genotyped on Affymetrix 6.0 and 565,040 autosomal SNPs after QC. We estimated the genetic relationships between all pairwise samples and removed one of each pair of samples with estimated genetic relationship > 0.025 and retained 11,586 unrelated samples.

For the univariate analysis, we randomly sampled 1000 SNPs as causal variants. The effect sizes of the causal variants were generated from standard normal distribution. We then calculated the total genetic value of an individual *i* as $g_i = \sum_j w_{ij} b_j$ where $w_{ij} = (x_{ij} - 2p_j) / \sqrt{2p_j(1-p_j)}$ with x_{ij} being coded as 0, 1 or 2 for the three genotypes of a causal variant *j* and p_j being the allele frequency, and b_j is the effect size of the causal variant *j*. We simulated the phenotype of each individual as $y = g + e$ where e was generated from a normal distribution with mean of 0 and variance of $\mathrm{var}(g)(1/h^2 - 1)$ with h^2 being the heritability. This procedure was performed using GCTA [5]. We simulated phenotypes under three different levels of heritability, i.e. 0.2, 0.5 and 0.8, and performed the simulations for a range of sample sizes, i.e. 1000, 1500, …, 5000, randomly sampled from the set of 11,586 unrelated individuals. We repeated the simulation 100 times in each scenario.

For bivariate analysis, we simulated the phenotypes using the same approach as in the univariate analysis. The phenotypes were simulated in two scenarios, I) the two simulated traits are "measured" on the same sets of individuals; II) the two

simulated traits are measured on different sets of samples. We simulated the genetic overlap (genetic correlation) between traits by choosing a proportion of the simulated causal variants to be common to both traits. We chose four levels of degrees of genetic overlap, i.e. 0%, 40% and 80% of causal variants in common. The effect sizes were fixed to be the same for these causal variants in common between the two traits. In scenario I where the two traits are measured on the same sample, we chose a range of sample sizes, i.e. 4000, 6000, 8000 and 10000. In scenario II where traits are measured on different sets of samples, we chose sample sizes of 1000, 2000, 3000 and 4000 for sample set #1 and 3000, 4000, 5000 and 6000 for set #2. The two traits were simulated based on heritability parameters of 0.4 and 0.6, respectively. We also repeated the simulations 100 times in each scenario.

We then used GCTA [5] to estimate the genetic variance or genetic correlation and its corresponding SE using all SNPs in each of the simulation scenarios.

References

1. Rimm EB, Giovannucci EL, Willett WC, Colditz GA, Ascherio A, et al. (1991) Prospective study of alcohol consumption and risk of coronary disease in men. Lancet 338: 464-468.
2. Colditz GA, Hankinson SE (2005) The Nurses' Health Study: lifestyle and health among women. Nat Rev Cancer 5: 388-396.
3. Psaty BM, O'Donnell CJ, Gudnason V, Lunetta KL, Folsom AR, et al. (2009) Cohorts for Heart and Aging Research in Genomic Epidemiology (CHARGE) Consortium: Design of prospective meta-analyses of genome-wide association studies from 5 cohorts. Circ Cardiovasc Genet 2: 73-80.
4. Yang J, Manolio TA, Pasquale LR, Boerwinkle E, Caporaso N, et al. (2011) Genome partitioning of genetic variation for complex traits using common SNPs. Nat Genet 43: 519-525.
5. Yang J, Lee SH, Goddard ME, Visscher PM (2011) GCTA: a tool for genome-wide complex trait analysis. Am J Hum Genet 88: 76-82.
