# Supplementary material for: Statistical Power to Detect Genetic (Co)Variance of Complex Traits Using SNP Data in Unrelated Samples
Source: PLoS Genet. 2014 Apr 10;10(4):e1004269. doi: 10.1371/journal.pgen.1004269 (PMC3983037; doi:10.1371/journal.pgen.1004269)
Supplement: Text S2 — Sampling variance of genetic correlation. (PDF) [file pgen.1004269.s004.pdf]

Text S2: Sampling variance of genetic correlation

Assume that two traits y_1 and y_2 are measured on all individuals with heritabilities of h_1^2 and h_2^2, genetic correlation of r_G, and phenotypic correlation of r_P. Assume that y_1 and y_2 are standardised so that $E(y_1) = E(y_2) = 0$ and $var(y_1) = var(y_2) = 1$. If we construct two new traits,

$z_1 = y_1 + y_2 = a_1 + e_1$ and $z_2 = y_1 - y_2 = a_2 + e_2$

where a_1 and a_2 are the genetic effects, we then have

$var(z_1) = 2(1 + r_P)$, $var(z_2) = 2(1 - r_P)$ and $cov(z_1, z_2) = 0$

$var(a_1) = h_1^2 + h_2^2 + 2h_1 h_2 r_G$, $var(a_2) = h_1^2 + h_2^2 - 2h_1 h_2 r_G$, and $cov(a_1, a_2) = h_1^2 - h_2^2$

If we denote h_{z1}^2 and h_{z2}^2 as the heritabilities for the two new traits, then

$h_{z1}^2 = var(a_1)/var(z_1)$ and $h_{z2}^2 = var(a_2)/var(z_2)$

We know from our derivations above that $var(\hat{h}_{z1}^2) = var(\hat{h}_{z2}^2) = 2/[N^2 var(A_{ij})]$, with *N* being the sample size and $var(A_{ij})$ being the variance of the genetic relationship between individuals *i* and *j*.

We can calculate r_G from the equations above

$r_G = [var(a_1) - var(a_2)]/[var(a_1) + var(a_2) - 2(h_1 - h_2)^2]$

Let $\alpha = var(a_1)$, $\beta = var(a_2)$ and $\Delta = 2(h_1 - h_2)^2$, then

$\hat{r}_G = [\hat{\alpha} - \hat{\beta}]/[\hat{\alpha} + \hat{\beta} - \hat{\Delta}]$

A first order Taylor approximation of the sampling variance of $\hat{r}_G$ is

$$var(\hat{r}_G) \approx \left(\frac{\partial \hat{r}_G}{\partial \hat{\alpha}}\right)^2 var(\hat{\alpha}) + \left(\frac{\partial \hat{r}_G}{\partial \hat{\beta}}\right)^2 var(\hat{\beta}) + 2\frac{\partial \hat{r}_G}{\partial \hat{\alpha}}\frac{\partial \hat{r}_G}{\partial \hat{\beta}} cov(\hat{\alpha}, \hat{\beta})$$

where the sampling variance of $\hat{\Delta}$ is ignored because $\hat{\Delta}$ is likely to be small.

Since $\frac{\partial \hat{r}_\mathrm{G}}{\partial \hat{\alpha}} = \frac{2\hat{\beta} - \hat{\Delta}}{(\hat{\alpha} + \hat{\beta} - \hat{\Delta})^2}$ and $\frac{\partial \hat{r}_\mathrm{G}}{\partial \hat{\beta}} = \frac{-2\hat{\alpha} + \hat{\Delta}}{(\hat{\alpha} + \hat{\beta} - \hat{\Delta})^2}$, we can get

$$\frac{\partial \hat{r}_\mathrm{G}}{\partial \hat{\alpha}} = \frac{2(\hat{h}_1^2 + \hat{h}_2^2 - 2\hat{r}_\mathrm{G}\sqrt{\hat{h}_1^2\hat{h}_2^2}) - 2(\hat{h}_1^2 + \hat{h}_2^2 - 2\sqrt{\hat{h}_1^2\hat{h}_2^2})}{16\hat{h}_1^2\hat{h}_2^2} = \frac{1 - \hat{r}_\mathrm{G}}{4\sqrt{\hat{h}_1^2\hat{h}_2^2}}$$

$$\frac{\partial \hat{r}_\mathrm{G}}{\partial \hat{\beta}} = \frac{-2(\hat{h}_1^2 + \hat{h}_2^2 + 2\hat{r}_\mathrm{G}\sqrt{\hat{h}_1^2\hat{h}_2^2}) + 2(\hat{h}_1^2 + \hat{h}_2^2 - 2\sqrt{\hat{h}_1^2\hat{h}_2^2})}{16\hat{h}_1^2\hat{h}_2^2} = \frac{-1 - \hat{r}_\mathrm{G}}{4\sqrt{\hat{h}_1^2\hat{h}_2^2}}$$

Since $\mathrm{cov}(z_1, z_2) = 0$, $\mathrm{cov}(\hat{\alpha}, \hat{\beta}) \approx 0$ so that

$$\begin{aligned}
\mathrm{var}(\hat{r}_\mathrm{G}) &\approx \left(\frac{1 - \hat{r}_\mathrm{G}}{4\sqrt{\hat{h}_1^2\hat{h}_2^2}}\right)^2 \mathrm{var}(\hat{\alpha}) + \left(\frac{1 + \hat{r}_\mathrm{G}}{4\sqrt{\hat{h}_1^2\hat{h}_2^2}^2}\right)^2 \mathrm{var}(\hat{\beta}) \\
&= \frac{(1 - \hat{r}_\mathrm{G})^2}{16\hat{h}_1^2\hat{h}_2^2}[\mathrm{var}(z_1)]^2 \,\mathrm{var}(\hat{h}_{z1}^2) + \frac{(1 + \hat{r}_\mathrm{G})^2}{16\hat{h}_1^2\hat{h}_2^2}[\mathrm{var}(z_2)]^2 \,\mathrm{var}(\hat{h}_{z1}^2) \\
&= \frac{(1 - \hat{r}_\mathrm{G} r_\mathrm{P})^2 + (\hat{r}_\mathrm{G} - r_\mathrm{P})^2}{\hat{h}_1^2\hat{h}_2^2 N^2 \,\mathrm{var}(A_{ij})}
\end{aligned}$$

For power calculation, we can substitute the estimates with the true parameters. We then have

$$\mathrm{var}(\hat{r}_\mathrm{G}) \approx \frac{(1 - r_\mathrm{G} r_\mathrm{P})^2 + (r_\mathrm{G} - r_\mathrm{P})^2}{h_1^2 h_2^2 N^2 \,\mathrm{var}(A_{ij})}$$
