# Supplementary material for: Statistical Power to Detect Genetic (Co)Variance of Complex Traits Using SNP Data in Unrelated Samples
Source: PLoS Genet. 2014 Apr 10;10(4):e1004269. doi: 10.1371/journal.pgen.1004269 (PMC3983037; doi:10.1371/journal.pgen.1004269)
Supplement: Text S3 — Acknowledgments to dbGaP data. (PDF) [file pgen.1004269.s005.pdf]

Text S3: Acknowledgments to dbGaP data

Funding support for the GWAS of Gene and Environment Initiatives in Type 2 Diabetes was provided through the NIH Genes, Environment and Health Initiative [GEI] (U01HG004399). The human subjects participating in the GWAS derive from The Nurses' Health Study (NHS) and Health Professionals' Follow-up Study (HPFS) and these studies are supported by National Institutes of Health grants CA87969, CA55075, and DK58845. Assistance with phenotype harmonization and genotype cleaning, as well as with general study coordination, was provided by the Gene Environment Association Studies, GENEVA Coordinating Center (U01 HG004446). Assistance with data cleaning was provided by the National Center for Biotechnology Information. Funding support for genotyping, which was performed at the Broad Institute of MIT and Harvard, was provided by the NIH GEI (U01HG004424).

The Atherosclerosis Risk in Communities Study (ARIC) is carried out as a collaborative study supported by National Heart, Lung, and Blood Institute contracts (HHSN268201100005C, HHSN268201100006C, HHSN268201100007C, HHSN268201100008C, HHSN268201100009C, HHSN268201100010C, HHSN268201100011C, and HHSN268201100012C), R01HL087641, R01HL59367 and R01HL086694; National HumanGenome Research Institute contract U01HG004402; and National Institutes of Health contract HHSN268200625226C. The authors thank the staff and participants of the ARIC study for their important contributions. Infrastructure was partly supported by Grant Number UL1RR025005, a component of the National Institutes of Health and NIH Roadmap for Medical Research.
